# HOFs Built from Hexatopic Carboxylic Acids: Structure, Porosity, Stability, and Photophysics

**DOI:** 10.3390/ijms23041929

**Published:** 2022-02-09

**Authors:** Maria Rosaria di Nunzio, Yuto Suzuki, Ichiro Hisaki, Abderrazzak Douhal

**Affiliations:** 1 Departamento de Química Física, Facultad de Ciencias Ambientales y Bioquímica, and INAMOL, Universidad de Castilla-La Mancha, Avenida Carlos III, S/N, 45071 Toledo, Spain; mrosaria.dinunzio@uclm.es; 2 Division of Chemistry, Graduate School of Engineering Science, Osaka University, Osaka 565-0871, Japan; y-suzuki@mac.chem.es.osaka-u.ac.jp

**Keywords:** hydrogen-bonded organic frameworks, hexatopic carboxylic acids, porosity, stability, time-resolved spectroscopy, photophysics

## Abstract

Hydrogen-bonded organic frameworks (HOFs) have attracted renewed attention as another type of promising candidates for functional porous materials. In most cases of HOF preparation, the applied molecular design principle is based on molecules with rigid π-conjugated skeleton together with more than three H-bonding groups to achieve 2D- or 3D-networked structures. However, the design principle does not always work, but results in formation of unexpected structures, where subtle structural factors of which we are not aware dictate the entire structure of HOFs. In this contribution, we assess recent advances in HOFs, focusing on those composed of hexatopic building block molecules, which can provide robust frameworks with a wide range of topologies and properties. The HOFs described in this work are classified into three types, depending on their H-bonded structural motifs. Here in, we focus on: (1) the chemical aspects that govern their unique fundamental chemistry and structures; and (2) their photophysics at the ensemble and single-crystal levels. The work addresses and discusses how these aspects affect and orient their photonic applicability. We trust that this contribution will provide a deep awareness and will help scientists to build up a systematic series of porous materials with the aim to control both their structural and photodynamical assets.

## 1. Introduction

Facing worldwide challenges such as sustainable energy, environmental remediation, and human welfare is at the vanguard of modern scientific research in the last 30 years. To this end, multifunctional porous materials including inorganic (silica-based materials, SBMs), covalent organic frameworks (COFs), and inorganic–organic hybrid types (metal–organic frameworks, MOFs) have been widely used as highly flexible platforms for the exploration of their potential use in diverse areas of science and technology such as (photo)catalysis [1,2,3,4,5,6,7,8,9,10,11], photonics [4,8,10,11], photo(sensing) [4,8,10,11,12], (opto)electronics [4,5,8,11,13,14,15], drug (photo)delivery [4,11,16], photo-chemotherapy [17], and medical imaging [13]. Recently, hydrogen-bonded organic frameworks (HOFs) also have acquired interest among scientists thanks to their potential in multidisciplinary fields. HOFs are crystalline materials with permanent porosity assembled thanks to H-bonding interactions between their molecular units, which can be pure organic or metal–organic moieties. The resulting frameworks can be further reinforced via additional weak interactions such as π–π interactions, van der Waals interactions, or CH–π interactions [18,19,20].

The milestone of H-bonded networked frameworks was trimesic acid (TMA), reported in 1969 [21]. The three-dimensional (3D) superstructure of hexagonal networks is based on neighboring TMA units linked by H-bonds among carboxyl groups. However, the interest in using H-bonds to assemble ordered networks was revived only in the late 1980s, when the crystal structure of inclusion complexes of TMA was determined [22], and the adamantane-1,3,5,7-tetracarboxylic acid (ADTA) crystallized to give a diamondoid network [23]. Later on, a large number of HOFs were characterized, and geometrically well-defined rigid organic molecules including pyridinone and diaminotriazine (DAT) were used to assemble a series of HOFs with predictable architectures, a guide strategy known as molecular tectonics [24,25,26,27,28,29,30,31,32,33,34,35,36,37,38,39,40,41]. Major advances came in the early 2010s, when the use of dipeptide crystals [42], pyridine [43], DAT [44], benzimidazolone [45], and pyrazole [46] led to the construction of HOFs with permanent porosity. This triggered further incentives to develop multifunctional HOFs. Moreover, the introduction of auxiliary π–π interactions to give more rigidity to the extended networks spawned considerable progress in realizing structurally stable HOFs with permanent porosities. Computational methods have been also used to additionally support the discovery of new HOFs with appropriate functions [47,48,49,50,51,52].

Typical aspects of HOFs [53,54], when compared with other porous materials such as MOFs and COFs, are: (1) high adaptability and reversibility of the H-bonds, which provide to HOFs intrinsic advantages such as simple synthesis conditions, good solution processability/regeneration, and easy recovery by recrystallization; (2) controlled synthesis, ensuring the cultivation and development of fine single crystals of HOFs, the structure determination of which is much easier compared to the case of MOFs, COFs, and SBMs; (3) lower density and toxicity than MOFs, thanks to the absence of heavy or transition metal species; (4) an ability to restore crystallinity by reannealing, and easy regeneration via a solution process; (5) greater stability toward water or humidity, differently from that of MOFs; (6) excellent flexibility, which can provide structural changes to generate functionality; and (7) the possibility to generate additional transient species by proton transfer (PT) reactions among the fundamental units. These special qualities make HOFs appealing materials for the development of related science and technology. Nevertheless, the H-bond reversibility also causes some problems. Firstly, HOFs tend to collapse during the activation process (i.e., the solvent removal from the voids). Secondly, the design of HOFs is not very easy, because sometimes the assembly of the molecular units leads to nonporous crystals instead of the desired, porous HOF. To solve this question, one of the key points is to merge other secondary intermolecular interactions, such as π–π interactions, with H-bonds to build stable porous HOFs. Another strategy is the creation of strong H-bonds such as charge-assisted H-bonds [55].

Nowadays, HOFs have several applications: for example, in gas separation and storage [43,44,56,57,58,59,60,61,62,63,64], heterogeneous catalysis [65,66,67], biomedicine [68,69], and proton conduction [70,71,72,73,74]. In addition, they have been proposed for photonic applications [54] such as sensing [75,76,77,78,79,80,81,82,83], photocatalysis [84,85], biomedicine [86], and optoelectronics [87]. From 2015, there has been a resurgence of interest in the use of homodimers of carboxyl groups as the H-bonding synthons for the assembly of HOFs, thanks to the large variety of geometries available for the carboxyl linkers [53,88]. For example, it has been reported that 1,3,5-tris(4-carboxyphenyl)benzene (**1**) resulted in stable HOFs based on interpenetrated honeycomb networks [89]. *C*_6_-symmetric hexacarboxylic acid **2** has been shown, on the other hand, to form a layered superstructure without interpenetration of 2D H-bonded networks [90]. Therefore, *C*_3_-symmetric π-conjugated molecules with six carboxy groups as building blocks for HOFs were used during the last six years for developing new HOFs. Recently, a C_2_H_2_ separable HOF composed of hexacarboxylic acid **3** was reported [91].

**Figure 1:**
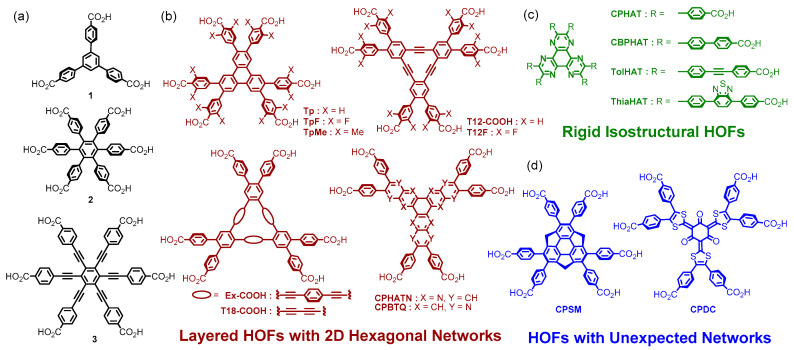
Examples of carboxylic acids for HOFs construction: (**a**) tri- and hexa-substituted benzene derivatives; (**b**) *C*_3_-symmetric π-conjugated molecules (*C*_3_PIs) providing layered HOFs; (**c**) *C*_3_PIs providing 3D-networked rigid HOFs; (**d**) *C*_3_PIs providing HOFs with unexpected H-bonded networks. The methyl ester derivatives corresponding to the carboxylic acids are denoted by the postfixed -COOMe (e.g., the ester derivative of T12-COOH is described as T12-COOMe).

This feature article focuses on the structure, porosity, stability, and photophysics of HOFs composed of hexatopic building block molecules, reported mainly by our groups. Hexatopic building blocks can provide robust frameworks with a variety of topologies and properties. The use of carboxyl groups can enable systematic construction of a series of HOFs as they are conceived from the starting design. The spectroscopy, photophysics, and related photonic applications of the HOFs described here are classified in three types of families, depending on their H-bonded structural motifs: (1) flexible HOFs formed by lamination of two-dimensional (2D) hexagonal networks; (2) rigid isostructural HOFs formed by interpenetration of 3D networks and shape-fitted molecular docking; and (3) HOFs with unexpected network structures (Figure 1).

We join in a unique work the synergic combination of experiences in HOFs’ chemistry and laser-based time-resolved (from femto-to-millisecond regime) spectroscopy and fluorescence microscopy techniques. There are two principal key factors that are unravelled in this feature article: (1) the chemical aspects that regulate both the chemistry and structures of the HOFs; and (2) their ensemble and single-crystal spectroscopy and photophysics at different time scales. This contribution also focuses on and considers in which way these aspects regulate the photonic applicability of these materials. Last, but not least, we pose questions and provide our vision for the next HOF generations regarding: (1) how to improve the design strategy, synthesis routes, and crystallization of HOFs; (2) how to increase their stability at ambient temperature; (3) how to control and enhance their response to light for real-world applications; and (4) how to design HOF-based composites involving known fluorescent and sensor dyes for photonic applications. We expect that this contribution will provide a deep awareness to build up a systematic series of porous materials, with the aim to control both their structural and photodynamical properties and boost advanced research in this field. 

## 2. HOFs Constructed through the H-Bonding of π-Conjugated Hexacarboxylic Acids

**Figure 2:**
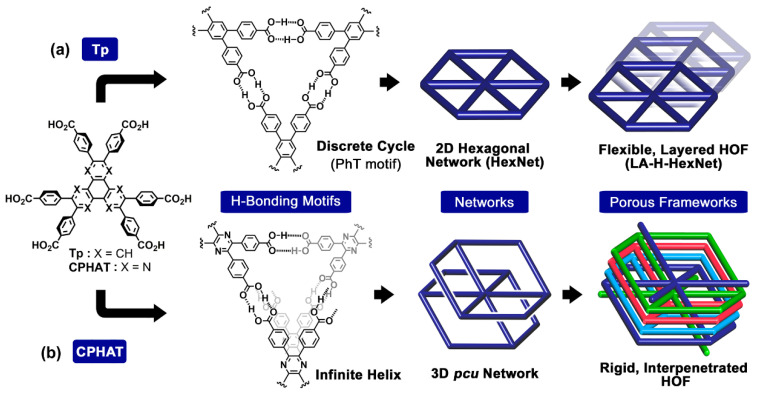
Hierarchical representations of (**a**) a flexible, layered HOF based on H-bonded hexagonal network and (**b**) a rigid, interpenetrated *pcu* framework, composed of *C*_3_-symmetric hexacarboxylic acids Tp and CPHAT, respectively.

The presence of carboxyl groups in the building block encourages the construction of geometrically well-defined frameworks thanks to the formation of highly directional H-bonded dimers of the carboxyl moieties. Carboxylic acid-based HOFs with permanent porosity have been intensively investigated since 2015 [53,92]. This section deals with the working hypothesis proposed few years ago by Hisaki’s group, with the aim to systematically build up HOFs with isostructural structure motifs. For this purpose, *C*_3_-symmetric π-conjugated planar building blocks (*C*_3_PIs) bearing three *o*-bis(4-carboxyphenyl)benzene groups in the periphery were chosen (Figure 2a). It has been proved that these units can generate isostructural 2D H-bonded hexagonal network (H-HexNet) sheets through H-connected carboxyl dimers, and that the H-HexNets further stack without interpenetration to provide flexible porous layered HOFs (LA-H-HexNets) [19,93,94,95,96]. The key structure for the formation of H-HexNets is the so-called phenylene triangle (PhT) motif, formed by the peripheral three *o*-bis(4-carboxyphenyl)benzene moieties. Furthermore, an unexpected result was achieved for a hexaazatriphenylene (HAT) derivative with 4-carboxyphenyl groups in the periphery, which formed no layered HOF (LA-H-HexNet) but a rigid 3D structured HOF [97]. This result was explained in terms of a twisted configuration assumed by the HAT core in the crystalline state, resulting in the formation of a 3D H-bonded network with primitive cubic (*pcu*) topology (Figure 2b). 

Below, we comment on the chemical, structural, and spectro-photodynamical properties of HOFs assembled using various *C*_3_PIs. In particular, the connection between structural and optical properties within homologous HOFs will be highlighted and discussed.

### 2.1. HOFs Based on Dehydrobenzo[12]annulene (DBAs) and Triphenylene Derivatives

**Figure 3:**
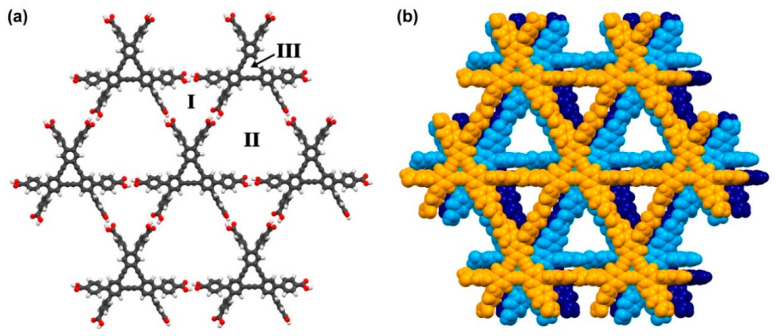
Crystal structure of T12-apo: (**a**) H-bonded HexNet structure; (**b**) LA-H-HexNet.

**Figure 4:**
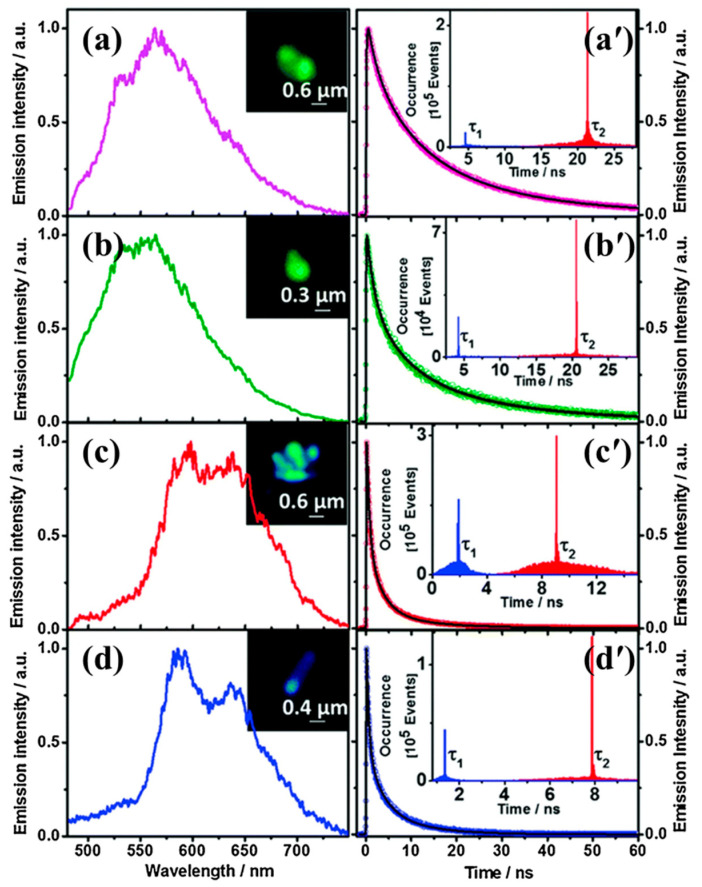
Emission (**a**–**d**) spectra and (**a′**–**d′**) decays of small T12-apo crystals. The insets in (**a**–**d**) show the fluorescence lifetime imaging (FLIM) images of the samples, and those in (**a′**–**d′**) give the lifetime distribution histogram. Reproduced with permission from the Royal Society of Chemistry, 2018.

The most important advantages of DBAs are planarity and high π-conjugation [98,99,100]. Depending on the molecular structure of the DBA unit and the number and kinds of π–π and H-bonding interactions, the resulting HOF will possess a specific morphology, crystallinity, and pore size [101]. Planar rigid tectons, such as T12-COOH (Figure 1), in which the peripheral carboxyl groups lay along the same molecular plane, form 2D hexagonal networked sheets that subsequently stack without interpenetration to form the corresponding layered T12-apo HOF (Figure 3) [19]. The HOFs deriving from the assembly of *C*_3_-symmetry-building blocks present two pores: (1) a narrower one (pore-I), corresponding to the triangular void space involving the PhT motif, with a constant side length of ~11 Å; and (2) a nonregular hexagonal-shaped wider one (pore-II), with longer (L = 15.8 Å) and shorter (r) sides. While L remains constant, r is affected by the size of the *C*_3_PI core. Specifically, T12-apo has an r value of 4.6 Å. Additionally, cyclic *C*_3_PI molecules can supply a third void (pore-III) due to the intrinsic pores within the molecules. However, pore-III of T12 is too narrow to accommodate a molecule. Activated HOF T12-apo was thermostable up to 360 °C and possessed permanent porosity with a Brunauer–Emmett–Teller surface area SA(BET) of 557 m^2^g^−1^ [19]. Both solid materials T12-apo and T12-ester, the latter being a solid bulk composed of T12-ester, exhibited a strong S_0_-S_1_ transition with intense absorption signals [102]. The use of single-molecule (crystal) fluorescence microscopy to study T12-ester crystals revealed different photodynamic behaviors depending on whether the investigated crystals had a large (>40 µm) or small (~0.5 µm) size, but while having very similar emission spectra [103]. Large crystals showed a monoexponential emission decay (~28 nanoseconds, ns), while small ones presented an extra component (~5 ns) assigned to the emission of species having suffered an intramolecular charge transfer (ICT) reaction from the methoxycarbonylphenyl groups to the unit core. Furthermore, for small (~0.3–1 µm) T12-apo crystals, the emission spectra clearly depended on their shape and size (Figure 4).

Figure 4a–d shows two kinds of spectra belonging to the emission of distinct crystals: (1) those based on π–π stacking, which have a photobehavior similar to that of small T12-ester crystals (lifetimes: 4.1–4.5 and 20–21 ns associated with the emission of ICT and not H-bonded species, respectively; see Figure 4a′,b′); and (2) crystals governed by H-bonding interactions between the fundamental units, which emit at lower energies and relax faster (1.4–1.5 and 8.5–8.8 ns) with respect to the crystals described in (1) (see Figure 4c′,d′). The shorter emission lifetimes of crystals described in (2) above showed the importance of H-bonds in their structures that might lead to PT reactions in the building blocks. In contrast, both the spectral and dynamical properties of larger (>30 µm) T12-apo crystals did not significantly change. A biexponential behavior of the emission decays was found with lifetimes of 4–6.5 and ~17–22 ns, attributed respectively to ICT and non-H-bonded species. Due to its Commission Internationale de l’Éclairage (CIE) coordinates (0.42, 0.55) and a high (25%) fluorescence quantum yield (Φ_F_), T12-apo might be used for the creation of a metal-free white light-emitting diode (WLED) after covering a blue LED with this HOF.

**Scheme 1:**
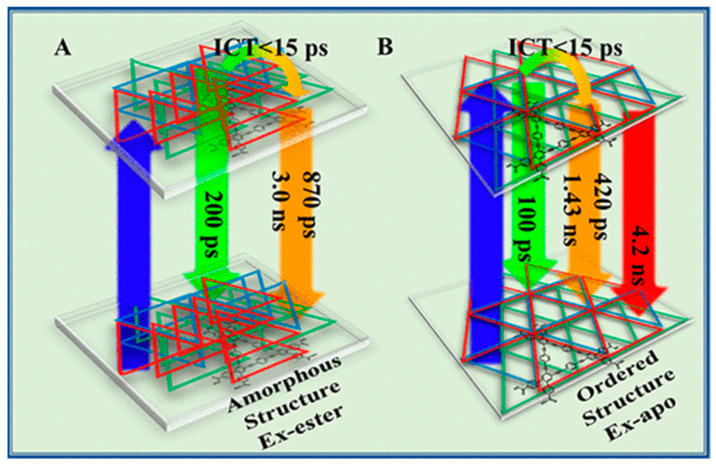
Schematic representation (not to scale) of the photodynamics of solid (**A**) Ex-ester and (**B**) Ex-apo showing the S_0_-S_1_ electronic transitions, involved processes, and relative time constants of the emission decays. The green, orange, and red arrows correspond to the fluorescence from LE, ICT, and anionic (only for the HOF Ex-apo) species, respectively. Reproduced with permission from the American Chemical Society, 2020.

Further expansion of the DBA structure gave birth to a new π-conjugated system, named the phenylene–ethynylene macrocycle, Ex-COOH or its methyl ester derivative (Ex-COOMe) (see Figure 1) [19,104]. In these frameworks, pore-II has an r value of 11.4 Å, which means that the pore has an almost regular hexagonal shape. Furthermore, pore-II has a diameter of 7.4 Å when the phenylene ring of the fundamental unit of Ex is perpendicular to the molecular plane. Ensemble behavior studies revealed a multiexponential emission decay of both solid samples of Ex-ester and Ex-apo, suggesting a heterogeneity of the systems. Emissions from locally excited (LE) and charge-transfer (CT) states were reported and discussed, with dynamics faster than those found in the parent T12-apo HOF (see above). In particular, the LE species displayed the shortest lifetimes (100–200 picoseconds (ps) for Ex-apo and Ex-ester, respectively), while those formed after fast ICT reactions (<15 ps) were slightly longer (420–870 ps and 1.43–3 ns for Ex-apo and Ex-ester, respectively) (see Scheme 1). Furthermore, an additional component of 4.20 ns in the emission decay was found for Ex-apo and was assigned to anions originating from strong H-bonding interactions between the Ex-COOH units.

Confocal fluorescence microscopy experiments on single crystals of Ex-apo disclosed no site-dependent photobehavior. Emission anisotropy experiments proved a good orientation of the emitters in the crystals.

**Figure 5:**
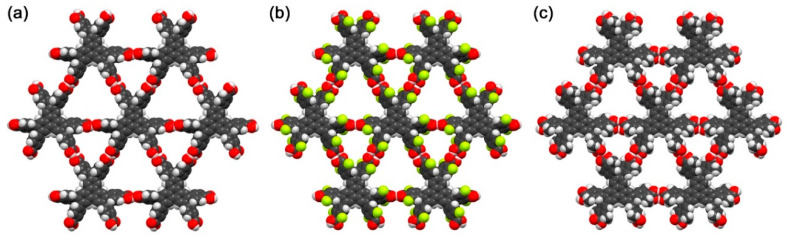
H-HexNet structures of: (**a**) Tp; (**b**) TpF; (**c**) TpMe. Crystallographically disordered parts in the frameworks are omitted for clarity.

Another *C*_3_PI unit possessing a good planarity and π-conjugation similar to the previously mentioned DBA-derivatives is the hexakis-(carboxyphenyl)triphenylene (Tp) unit (Figure 1), which is responsible for the formation of a dual-pored H-HexNet. Flexible layered 2D HOFs (LA-H-HexNets) with permanent porosity are then formed by the assembly of several H-HexNet sheets (Figure 5). Due to a distorted conformation assumed by the PhT molecular units, these structures show a good solubility and provide shape-persistent pores [45,105]. By introducing methyl (Me) or an F atom at the *o*-positions of the carboxyphenyl groups, two new compounds, named hexakis(4-carboxy-3,5-dimethylphenyl)triphenylene (TpMe) and hexakis(4-carboxy-3,5-difluorophenyl)triphenylene (TpF), were obtained (Figure 1) [106]. Substitution had no effect on the binding energy of the H-bonded dimerization, which was ~15 kcal mol^−1^ for all the obtained materials. On the other hand, Me and F substituents caused a twisting of the carboxyl and phenylene groups, thus increasing the flexibility of the peripheral conformation. Activation, defined as the desolvation with retaining pores, of LA-H-HexNets of TpMe and TpF leads to the formation of crystalline TpMe-apo and TpF-apo, respectively. Upon desolvation, both emission and excitation intensity maxima of TpMe-2Ds (TpMe-LA-H-HexNets before the activation) were shifted toward lower energies, indicating that TpMe-apo experiences stronger intermolecular interactions than TpMe-2Ds. A similar behavior was observed for TpF-1 (the TpF-LA-H-HexNets before the activation).

### 2.2. HOFs Based on Hexaazatriphenylene (HAT) and -Naphthylene (HATN) Derivatives

The use of heterocyclic π-conjugated compounds such as hexaazatriphenylene (HAT) derivatives leads to the formation of significantly robust HAT-based HOF systems thanks to the rigid, planar, and π-conjugated skeleton of their building blocks. The H-bonded dimers of carboxyl groups have the leading roles in the formation of the supramolecular architecture of these HOFs.

**Figure 6:**
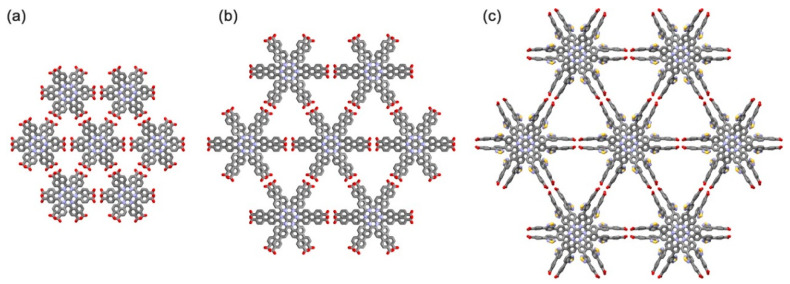
Crystal structures of HAT-based HOFs: (**a**) CPHAT-1; (**b**) CBPHAT-1; (**c**) ThiaHAT-1.

The first case of a HAT-based HOF presenting permanent porosity was reported in 2017. The fundamental unit was a HAT bearing six peripheral carboxyphenyl groups (CPHAT, Figure 1), which formed a three-dimensional (3D) framework with permanent porosity (CPHAT-1-(TCB = 1,2,4-trichlorobenzene)) (see Figure 6) [97]. The activation of CPHAT-1-(TCB) led to the corresponding CPHAT-1a, which preserved both permanent porosity and single-crystallinity. The pore’s size in this HOF was 6.4 Å, with an SA(BET) of 649 m^2^g^−1^. The larger π-conjugation of CPHAT generated by the presence of the phenyls and carboxylic groups was responsible for the redshift of its absorption spectrum with respect to that of pristine HAT. Considering that the CPHAT moieties are rotated by 60°, this arrangement can hinder the hopping of charge-carrier species [107]. This suggestion was then confirmed by flash-photolysis time-resolved microwave (FP-TRMC) measurements of CPHAT-1-(TCB) and CPHAT-1a crystals, which revealed no charge transport ability [108,109]. CPHAT-1-(TCB) and CPHAT-1a crystals exhibited low emission quantum yields (Φ_F_) due to efficient nonradiative deactivations triggered by strong stacking molecular interactions. Single-molecule fluorescence microscopy experiments indicated highly anisotropic emission behaviors, reflecting that both CPHAT-1-(TCB) and CPHAT-1a possess ordered crystalline structures, with molecular dipole moments oriented perpendicularly with respect to the long crystal axis. Moreover, CPHAT-1-(TCB) presented a heterogeneous distribution of the molecular interactions due to the presence of TCB, while for CPHAT-1a, the distribution was very homogeneous, with the units forming the crystal having similar interactions.

Addition of three benzenes at the HAT unit led to the corresponding carboxyphenyl-substituted hexaazatrinaphthylene derivative (CPHATN) as the fundamental unit (Figure 1) [79].

**Figure 7:**
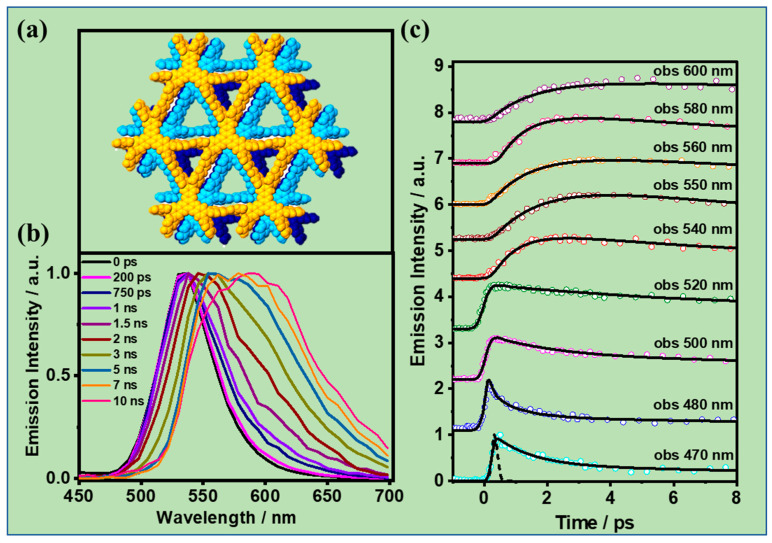
(**a**) Framework of CPHATN-1a. (**b**) Ps-time-resolved emission spectra and (**c**) femtosecond (fs)-emission transients of CPHATN-1a crystals. The observation wavelengths and gating times are indicated in the insets. The dashed line in (**c**) is the instrumental response function (IRF).

**Figure 8:**
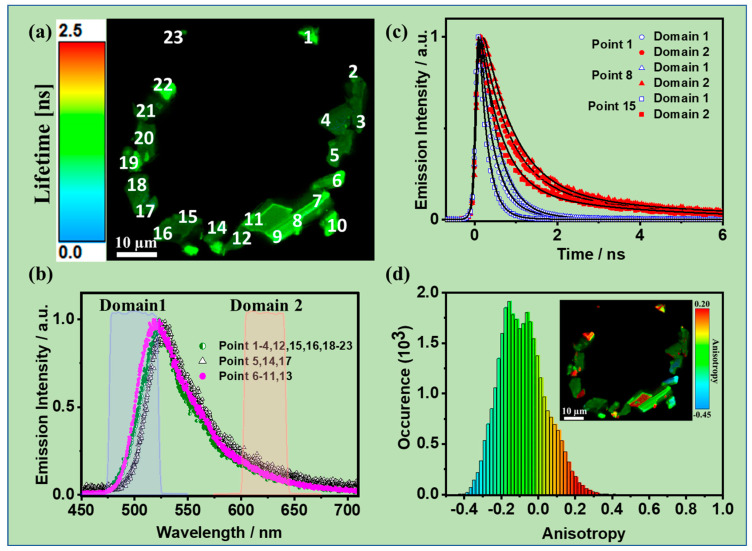
(**a**) FLIM of CPHATN-1a crystals. (**b**) Emission spectra of the crystals (or sites) shown in (**a**). (**c**) Emission decays at selected spectral ranges as indicated in (**b**). (**d**) Histogram of the emission anisotropy of the crystals (inset). Reproduced with permission from the American Chemical Society, 2019.

This chemical modification had the aim to boost both the electronic and sensing capability of CPHAT-1a. The H-HexNet resulted from the union of the CPHATN building blocks further assemble to result in a layered HOF (Figure 7a), the activated form of which was named CPHATN-1a. The latter had a pore diameter of 7.8 Å, which was larger than the one measured for CPHAT-1a (6.4 Å). Moreover, the three extra benzenes conferred to the core of CPHATN-1a a greater π-conjugation with respect to the case of CPHAT-1a; this resulted in redshifted steady-state absorption and emission spectra of the former compared to the latter. The difference among the excitation and absorption spectra suggested the existence of efficient nonradiative relaxation channels due to the strong coupling of the unpaired core nitrogen electrons with the (n-π*) states. Time-resolved (from fs to ps) experiments proved the existence of photoinduced ICT (ultrafast; ≤100 fs) and intermolecular proton transfer (iPT, fast; 1.1 ps) reactions involving the phenyl groups and the main core in the case of ICT, or the acid groups of the fundamental units of the crystals in the case of iPT (Figure 7b,c). The high anisotropic emission performance (short-width histogram of the emission anisotropy) of a CPHATN-1a single crystal demonstrated a preferential orientation of the molecular dipole moments perpendicular to the long axis with the π–π stacking. The main crystal anisotropy value (−0.44) was accompanied by a second one centered at −0.2, which was assigned to a minor population of the smallest crystals adsorbed on the surface of the larger one and possible defects. The ensemble anisotropy histogram presented a larger width compared to that of the histogram for the single crystal, plainly revealing the existence of differently oriented crystals (Figure 8).

**Figure 9:**
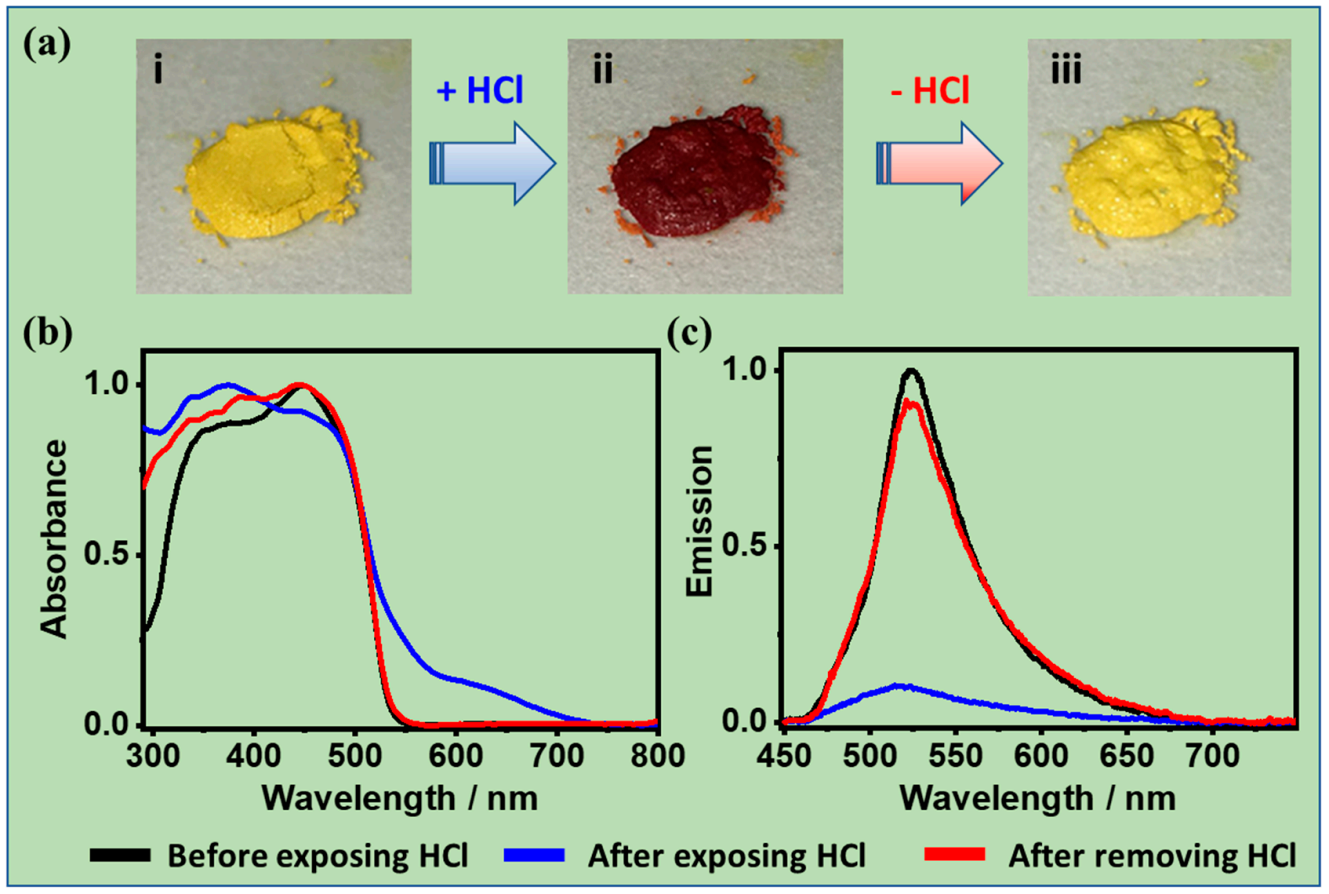
(**a**) Pictures showing the color changes of crystalline bulks of CPHATN-1a (**i**) before and (**ii**) after exposure to 37%-HCl, and (**iii**) after heating at 150 °C for 30 min. (**b**) Absorption and (**c**) emission spectra of solid CPHATN-1a upon exposure to HCl atmosphere for 40 min and after equilibrating the sample with ambient air for 48 h. Adapted with permission from the American Chemical Society, 2019.

Remarkably, CPHATN-1a has been shown to be an efficient and reversible sensor for HCl vapors [79]. Under HCl-saturated atmosphere, a clear color change (from yellow to reddish brown) was observed only in the solid-state as the consequence of the HATN core protonation and additional intermolecular interactions between protonated and neutral HATN units (Figure 9(ai,aii)). The pristine color was practically recuperated after heating at 150 °C for 30 min (Figure 9(aiii)). The absorption spectrum of acidified CPHATN-1a revealed a new band at 500–600 nm (Figure 9b), while the emission signal was strongly reduced (Figure 9c). Moreover, by removing the HCl exposure and clearing the crystals in air at ambient temperature for 48 h, 90% of both the absorption and emission intensities were recovered (Figure 9b,c).

**Figure 10:**
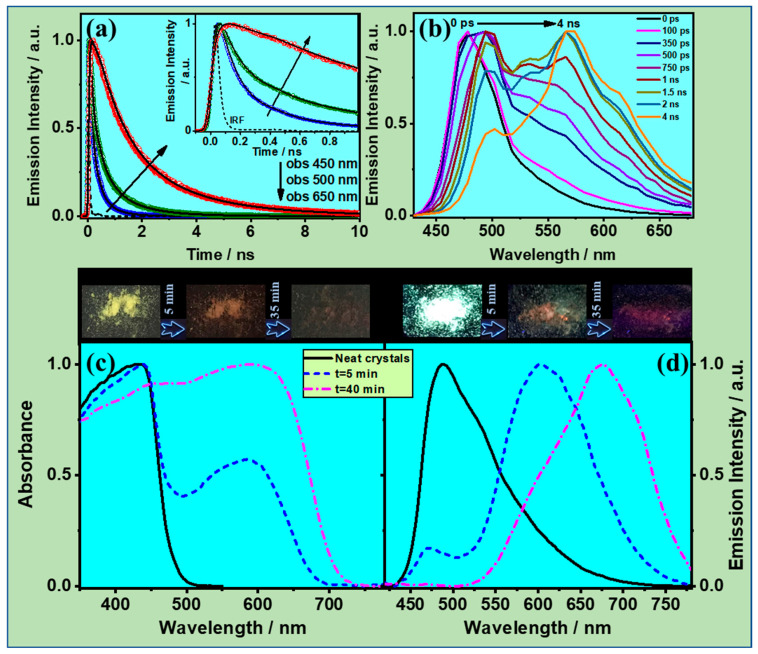
(**a**) Emission decays of solid CBPHAT-1a, recorded at the indicated wavelengths. The dashed signal is the IRF of the setup. (**b**) Normalized TRES of CBPHAT-1a in the solid-state at the indicated gating time. (**c**) Absorption and (**d**) emission spectra of CBPHAT-1a crystals before and after exposure to HCl atmosphere. The upper pictures in (**c**,**d**) show the color of the crystals before and after interaction with HCl atmosphere in ambient light and upon irradiation with a 360 nm lamp.

The structure of CPHAT was also modified by adding another benzene ring to each carboxyphenyl group, thus obtaining a HAT bearing six peripheral carboxybiphenyl groups (CBPHAT, Figure 1) [110]. Remarkably, this HOF presented pore (12.4 Å) and SA(BET) (1288 m^2^g^−1^) values of twice those corresponding to CPHAT-1. The solid-state UV–visible absorption spectra of CBPHAT-1a (Figure 6) and its butylated derivative (CBPHAT-C_4_H_9_) were quite similar, consisting of broad bands indicative of the presence of different ground-state species. On the other hand, the fluorescence spectrum of CBPHAT-1a showed an additional emission assigned to anions produced by strong H-bonding interactions between the networking HOF’s units. Figure 10a,b exhibits the ps-emission decays and time-resolved emission spectra (TRES) of solid CBPHAT-1a HOF. An ICT of 60 ps time constant was observed in the excited CBPHAT-1a molecules, and which was decaying within 380 ps. Two different populations, relaxing at 1.02 and 2.66 ns, were produced after the ICT process. The recorded TRES of CBPHAT-1a proved the formation of a new emission band at 566 nm at longer times (>350 ps) (Figure 10b), assigned to anions. As in the case of CPHATN-1a, the CBPHAT-1a crystal was HCl-responsive (Figure 10c,d). The new absorption band at ~600 nm suggested a robust interaction of the nitrogen atoms of the HAT core with the acid protons. Moreover, the new band intensity increased with the exposure time. The color change could be easily appreciated by the naked eye (upper pictures in Figure 10c,d). Once in contact with HCl vapors, the emission spectrum of CBPHAT-1a shifted toward the red (Figure 10d) due to a protonation of the nitrogen sites of the core of CBPHAT-1a. Remarkably, the CBPHAT-1a/HCl interaction (adsorption, desorption) was reversible even at ambient temperature.

Modification of the arms of CBPHAT-1a with 1,2-diphenylethyne and 4,7-diphenylbenzo-2,1,3-thiadiazole resulted in the formation of more expanded HOFs (TolHAT-1 and ThiaHAT-1) [111]. Particularly, ThiaHAT-1 (Figure 6) showed a great stability up to 305 °C and a high BET surface area of 1394 m^2^g^−1^, with a pore diameter of 15.5 Å. The photophysical properties of Tol-HAT-1 and ThiaHAT-1 were elucidated by steady-state and time-resolved spectroscopy, as well as single-crystal fluorescence microscopy. While TolHAT-1 was not stable under UV light excitation, probably due to the presence of the ethynyl moieties in the fundamental unit, ThiaHAT-1 exhibited an outstanding stability under light irradiation. Crystals of ThiaHAT-1 showed two broad absorption bands centered at ~435 and 600 nm together with a bright yellow emission upon 365 nm-illumination, with a Φ_F_ of 8%.

**Figure 11:**
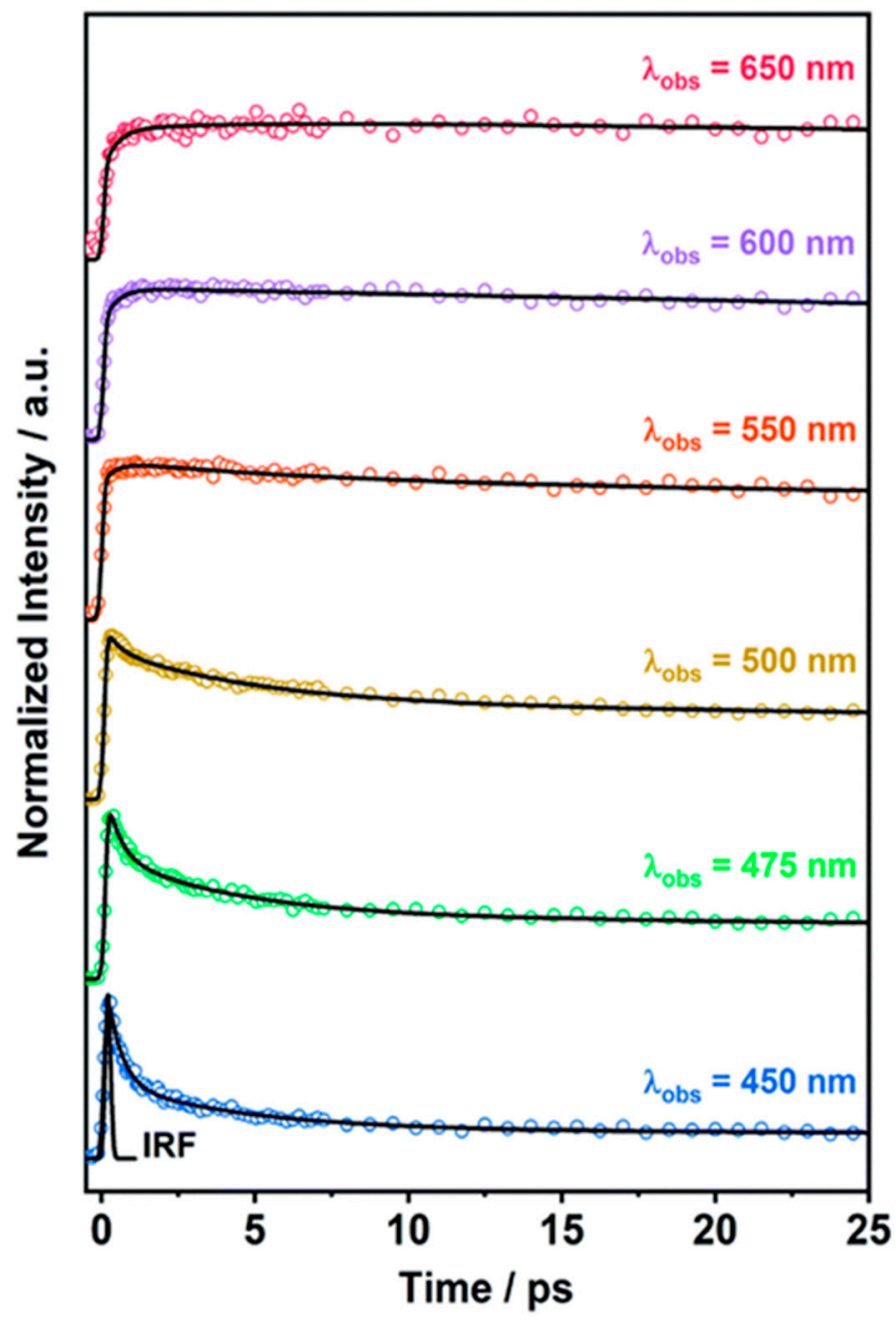
Fs-emission decays of ThiaHAT-1 in DMF suspension. The solid lines are from the best multiexponential global fit, and the IRF is the instrumental response function. This is an open access article under the CC BY-NC-ND license (http://creativecommons.org/licenses/by-nc-nd/4.0, accessed on 14 January 2022).

**Figure 12:**
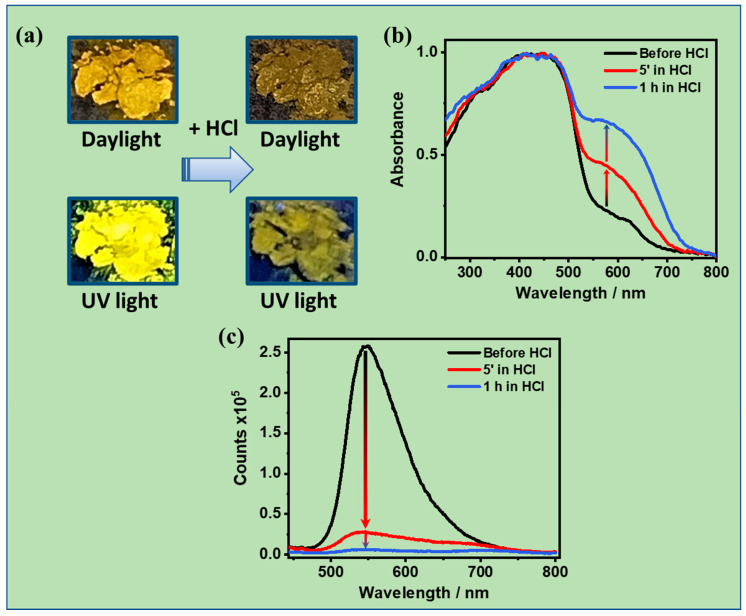
Acid responsiveness of ThiaHAT-1. (**a**) Photographs of the crystalline powder before (left) and after (right) being exposed to HCl under daylight (top) and UV light (365 nm, bottom). (**b**) Absorption and (**c**) emission spectra of ThiaHAT-1 before and after being exposed to vapors of HCl.

Time-resolved ps studies under 371 and 515 nm excitation revealed comparable multiexponential photobehavior, with lifetimes of 160–180 ps, 710–720 ps, and 2.3–2.5 ns. These components were respectively assigned to the emissions of LE, CT, and anionic species, similar to what was observed for the isostructural CBPHAT-1. Fs-emission experiments on ThiaHAT-1 in a DMF suspension allowed us to determine the time constants for the excited-state PT (4.4 ps) and CT (450 fs) reactions (Figure 11). ThiaHAT-1 also exhibited an excellent response to the presence of HCl vapors, and which could be easily observed by the naked eye under daylight or UV irradiation. The exposure of this HOF to HCl vapors for short times (5 min to 1 h) led to a strong bright yellow-to-dark-brown color change (Figure 12a). The ThiaHAT-1-HCl interaction could be clearly appreciated in both the absorption and emission spectra of the investigated HOF (Figure 12b–d). The additional emission band at 700 nm recorded in the presence of HCl reflected the protonation of ThiaHAT-1. Interestingly, ThiaHAT-1 preserved the dark brown color and quenched emission after the HCl treating, thus making this HOF a promising candidate for the construction of an HCl vapochromic smart sensor.

### 2.3. HOFs with Unusual H-Bonding Topology

In the previous sections, we highlighted the importance of highly symmetric planar π-conjugated cores that, armed with directional H-bonding interactions, can reasonably provide well-defined, 2D-networked porous sheets that then pile into crystalline layered frameworks. However, 2D frameworks made of nonplanar π-conjugated molecules are likewise interesting constructions. Sheets with bent and/or bumpy surfaces are in fact supposed to possess exclusive electronic, chemical, or physical properties due to the curved π-systems [112,113,114,115]. Nowadays, it is still a stimulating challenge to develop such new classes of functional materials. Basing on these considerations, we proposed the creation of H-bonded 2D frameworks by utilizing the *C*_3_-symmetric buckybowl, sumanene [116,117,118], which presents a distorted triphenylene moiety, and thus is an appropriate system to compare to our previous system of Tp HexNet frameworks [94]. The H-bonded 2D architecture of the buckybowl hexakis(carboxyphenyl)sumanene derivative CPSM provides two kinds of H-bonded 2D HexNet frameworks: (1) a waved HexNet structure with *hxl* (*hxl* = hexagonal lattice) network topology composed of alternate alignment of up- and downward bowls (CPSM-1); and (2) a bilayered HexNet structure (CPSM-2) in which all of the six carboxy groups contribute to form H-bonds to give hamburger-shaped sumanene dimers. Furthermore, CPSM-2 crystals underwent highly anisotropic shrinking along the *c* axis by about 11% after applying a uniform hydrostatic pressure of 970 MPa. This shrinking was caused by interlayer slithering of the bilayered HexNet sheets along the curved sumanene surfaces. This behavior can provide new understanding of 2D-frameworks built from nonplanar π-conjugated systems.

To continue with another example of an HOF with unusual topology, we recently reported the first 3D-networked HOF based on a carboxyphenyl-substituted tri(dithiolylidene)cyclohexanetrione (DC) derivative, CPDC [119]. The related HOF, CPDC-1, is assembled through an anomalistic helical H-bonded motif, instead of the conventional planar or simple helical motifs. This unusual H-bonding motif probably originates from a slightly larger bite angle (*θ*) of CPDC. As a consequence, CPDC-1 forms a robust 3D, non-interpenetrated network that preserves its stability up to 372 °C. The absorption spectrum of the ester derivative exhibited three mean peaks at 246, 370, and 470 nm, the latter displaying the maximum intensity. The hexabenzoate showed a 491 nm-centered emission band (exciting at 370 nm) with a relative weak Φ_F_ of 0.76%. Additionally, the highly π-delocalized redox core of CPDC promoted intermolecular π-stacking and S-S interactions (S refers to each sulfur atom in the dithiolylidene ring), supporting charge conduction in the HOF. From the absorption edge at 488 nm, an optical band gap of 2.54 eV was determined. Differential pulse voltammetry (DPV) experiments were carried out, aiming to define the redox behavior of the ester. The results indicated that all the three dithiole rings underwent sequential oxidation and reduction reactions. The HOMO and LUMO energies were found to be −5.82 and −3.57 eV, respectively, providing an electrochemical band gap of 2.25 eV. Theoretical calculations performed on CPDC and a methyl ester of CPDC revealed stabilization of the HOMO by electron-withdrawing carboxyl/carboxylate substituents.

## 3. Conclusions and Outlooks

In this feature article, we showed and commented on the latest progress and findings achieved by our groups on the use of *C*_3_-symmetric π-conjugated molecules (*C*_3_PIs) possessing three *o*-bis(4-carboxyphenyl)aryl moieties in the periphery for systematic construction of various HOFs. Planar *C*_3_PIs gave birth to isostructural 2D H-bonded hexagonal network (H-HexNet) sheets via H-bonded carboxyl dimers. These isostructural layers were created thanks to the so-called phenylene triangle (PhT), which is a triangular H-bonded motif. Additional non-interpenetrating stacking of the H-HexNets led to flexible porous layered HOFs (LA-H-HexNets). On the other hand, nonplanar *C*_3_PIs made feasible the construction of rigid 3D-networked HOFs. In particular, hexaazatriphenylene (HAT) cores formed a π-stacked column through shape-fitted docking, giving more rigid frameworks. Interpenetration of H-bonded networks also promoted the development of rigid HOFs. Interestingly, bowl-shaped *C*_3_PIs produced H-bonded networked structures with unusual network topology, in which all the carboxy groups were involved in the formation of H-bonded dimers. Furthermore, the use of a carboxyphenyl-substituted tri(dithiolylidene)cyclohexanetrione (DC) derivative, CPDC, also originated an HOF with an uncommon network topology. These exotically structured HOFs presented exclusive electronic, chemical, and photophysical properties. Hexatopic building blocks (i.e., with six H-bonding functional groups) can give robust frameworks with a large variety of topologies and properties. The use of carboxyl groups and simple high directional H-bonds as the molecular glue can empower the systematic construction of a series of HOFs. We join together in a unique work the experiences in HOFs’ chemistry and laser-based time-resolved (in the fs-to-ms regime) spectroscopy and microscopy techniques. The HOFs showed ultrafast and slow photoinduced events, ranging in the fs-to-ms regime, and reflecting, for example, ICT, strong H-bond interactions, iPT. We emphasized two main aspects, chemical and photophysical. The latter unravelled at both the ensemble and single-crystal levels. We also referred to and examined how these aspects influenced the photonic applicability of the studied HOFs. The future of HOFs is going to be reliant on a series of key challenges that must be solved; more specifically: (1) improving the design approach and synthesis routes and crystallization of HOFs; (2) increasing their stability at ambient temperature; (3) regulating and boosting their light response for real-world applications; and (4) making feasible the design of HOF-based composites involving known fluorescent and sensor dyes for photonic applications. Computational methods are also an important tool to additionally support the breakthrough of HOFs with appropriate functions.

## Abbreviations

HOF	Hydrogen-Bonded Organic Frameworks
2D	Two-Dimensional
3D	Three-Dimensional
SBMs	Silica-Based Materials
COFs	Organic Covalent Organic Frameworks
MOFs	Metal–Organic Frameworks
PT	Proton Transfer
TMA	Trimesic Acid
ADTA	Adamantane-1,3,5,7-Tetracarboxylic Acid
DAT	Diaminotriazine
*C*_3_PIs	*C*_3_-Symmetric π-Conjugated Planar Building Blocks
H-HexNet	H-Bonded Hexagonal Network
LA-H-HexNet	Layered H-Bonded Hexagonal Network
PhT	Phenylene Triangle
HAT	Hexaazatriphenylene
DBA	Dehydrobenzo[12]annulene
BET	Brunauer–Emmett–Teller
SA	Surface Area
Ns	Nanosecond
ICT	Intramolecular Charge Transfer
FLIM	Fluorescence Lifetime Imaging
CIE	Commission Internationale de l’Éclairage
LED	Light-Emitting Diode
WLED	White Light-Emitting Diode
LE	Locally Excited
CT	Charge Transfer
Ps	Picosecond
Tp	Hexakis-(Carboxyphenyl)Triphenylene
Me	Methyl
TpMe	Hexakis(4-Carboxy-3,5-Dimethylphenyl)Triphenylene
TpF	Hexakis(4-Carboxy-3,5-Difluorophenyl)Triphenylene
HAT	Hexaazatriphenylene
HATN	Hexaazatrinaphthylene
CPHAT	Carboxyphenyl Hexaazatriphenylene
TCB	1,2,4-Trichlorobenzene
FP-TRMC	Time-Resolved Microwave
CPHATN	Carboxyphenyl-Hexaazatrinaphthylene
Fs	Femtosecond
IRF	Instrumental Response Function
CBPHAT	Carboxybiphenyl Hexaazatriphenylene
TRES	Time-Resolved Emission Spectra
UV	Ultraviolet
vis	Visible
DMF	Dimethylformamide
CPSM	Hexakis(Carboxyphenyl)Sumanene
*hxl*	Hexagonal Lattice
DC	Tri(Dithiolylidene)Cyclohexanetrione
CPDC	Carboxyphenyl-Tri(Dithiolylidene)Cyclohexanetrione
DPV	Differential Pulse Voltammetry
HOMO	Highest Occupied Molecular Orbital
LUMO	Lowest Unoccupied Molecular Orbital
